# G-protein coupling and nuclear translocation of the human abscisic acid receptor LANCL2

**DOI:** 10.1038/srep26658

**Published:** 2016-05-25

**Authors:** Chiara Fresia, Tiziana Vigliarolo, Lucrezia Guida, Valeria Booz, Santina Bruzzone, Laura Sturla, Melody Di Bona, Mattia Pesce, Cesare Usai, Antonio De Flora, Elena Zocchi

**Affiliations:** 1 Department of Experimental Medicine, Section of Biochemistry, University of Genova, Italy; 2 Istituto Italiano di Tecnologia (IIT), Genova, Italy; 3 Institute of Biophysics, National Research Council (CNR), Genova, Italy.

## Abstract

Abscisic acid (ABA), a long known phytohormone, has been recently demonstrated to be present also in humans, where it targets cells of the innate immune response, mesenchymal and hemopoietic stem cells and cells involved in the regulation of systemic glucose homeostasis. LANCL2, a peripheral membrane protein, is the mammalian ABA receptor. We show that N-terminal glycine myristoylation causes LANCL2 localization to the plasmamembrane and to cytoplasmic membrane vesicles, where it interacts with the α subunit of a G_i_ protein and starts the ABA signaling pathway via activation of adenylate cyclase. Demyristoylation of LANCL2 by chemical or genetic means triggers its nuclear translocation. Nuclear enrichment of native LANCL2 is also induced by ABA treatment. Therefore human LANCL2 is a non-transmembrane G protein-coupled receptor susceptible to hormone-induced nuclear translocation.

The human genome encodes three distinct LANCL proteins, LANCL1, LANCL2 and LANCL3^1^, which share a high structural homology with the lanthionine synthetase component C, a cyclase involved in the synthesis of lantibiotics in Prokaryotes^2^. LANCL3 has been suggested to be a pseudogene^3^. LANCL1 has been hypothesized to be implicated in the metabolism of lanthionine metabolites in the central nervous system^4^. LANCL2 proved to be the human receptor of abscisic acid (ABA)^5^,^6^,^7^,^8^,^9^,^10^.

ABA, a long-known plant hormone^11^,^12^, has been shown to be present also in mammals, where it affects several key functions in different cell types^9^,^10^,^13^,^14^. ABA behaves as a pro-inflammatory modulator of cells of the innate immune response^7^,^15^,^16^,^17^, stimulates the proliferation of human mesenchymal and hemopoietic stem cells^18^,^19^, and is involved in the control of systemic glucose homeostasis^5^,^20^,^21^,^22^,^23^,^24^.

LANCL2-mediated ABA signaling in mammals requires a pertussis toxin (PTX)-sensitive G protein^5^, eventually leading to an increase of intracellular Ca^2+^ levels ([Ca^2+^]_i_). The signaling pathway downstream of ABA binding to LANCL2 involves the activation of adenylate cyclase (AC), followed by overproduction of cAMP, PKA-catalyzed phosphorylation and stimulation of the plasmamembrane-bound ADP-ribosyl cyclase CD38, which converts NAD^+^ to cADPR and ADPR, leading to an increase of both extracellular Ca^2+^ entry and Calcium-induced calcium release (CICR)-mediated intracellular Ca^2+^ mobilization^5^,^9^,^15^,^21^.

Several indirect lines of evidence point to a G_i_ as the G protein coupled to LANCL2: i) the sensitivity of the ABA signaling pathway to PTX in human granulocytes and in insulin-releasing cells^15^,^21^; ii) the accumulation of inositol 1,4,5-P3 (IP_3_) in human cells co-transfected with LANCL2 and a chimeric G protein, Gα_q/i_, upon stimulation with ABA^5^, and, iii) inhibition of the ABA-induced cAMP increase in ABA-sensitive human cells by overexpression of transducin, a βγ-subunit scavenger^5^. However, conclusive identification of the nature of the G protein coupled to LANCL2 has yet to be provided. For instance, the role of G_βγ_ in AC signaling is exceedingly complex, as witnessed by both AC-activating and inhibiting effects related to wide heterogeneity of the coupling receptors and of the various membrane-associated AC isoforms^25^,^26^. Moreover, the mechanism of the LANCL2-G protein coupling, specifically whether it is direct or mediated by other proteins, remains to be defined.

Interestingly, LANCL2 is not a transmembrane protein, as predicted *in silico* from its sequence^27^,^28^,^29^ and confirmed *in vitro* by the observation that it can be removed from the plasmamembrane without the use of detergents, either by mild chemical treatments^30^ or by inhibition of its post-translational N-terminal myristoylation^28^. In addition, the non-myristoylated LANCL2-GFP fusion protein has been found to be confined to the nucleus^28^. This observation raises the possibility that its hormone ligand ABA may affect the trafficking of LANCL2 between membranes and nucleus.

Indeed, recent findings allow to reconcile the non-transmembrane localization of LANCL2 with its hormone receptor function, as the human anion exchanger AE1 has been shown to mediate ABA transport across the plasmamembrane^30^.

Here, we investigated the unusual features of LANCL2 among G protein-coupled animal hormone receptors (GPCR), by means of site-directed mutagenesis and of confocal fluorescence microscopy, fluorescence recovery after photobleaching (FRAP) and photoactivation techniques. The localization, the intracellular mobility of LANCL2 in the presence of ABA and its interaction with G_i_ were explored.

## Results

### Role of N-terminal myristoylation in the subcellular localization of untagged LANCL2

Comparison between the three LANCL genes shows that Met 19 of LANCL2 is aligned with the start methionine of the highly homologous LANCL1 and LANCL3 (Supplementary Table 1), which are cytosolic proteins^27^,^28^,^29^. Thus, transcription of LANCL2 starting from Met 19 would result in a shorter protein, lacking the myristoylated glycine and the 18-aminoacids N-terminal stretch (LANCL2sh).

Supplementary Fig. 1 shows a representative Western blot of full length LANCL2 from HeLa, HEK and the human adenocarcinoma cell line MDA-MB-468 (MDA-468), revealed with a specific mAb (see Materials and Methods). In this cell line, a 0.9 Mb region, containing the LANCL2 gene, is amplified^31^. Overexpression of LANCL2 in MDA-468 cells was documented by microarray profile analysis, by RT-PCR and by measuring [^3^H]ABA binding to intact cells, in comparison with the MDA-MB-231 (a breast adenocarcinoma cell line not overexpressing LANCL2)^32^. The B_max_ values calculated from the relevant saturation curves by Scatchard plot analysis were 0.70 pmol ABA/mg protein for MDA-468 and 0.023 pmol ABA/mg protein for MDA-231, respectively.

Landlinger *et al*.^28^ demonstrated that GFP-fused LANCL2 overexpressed in human amniotic epithelial cells was associated with the plasmamembrane; moreover, inhibition of myristoylation either by mutation of the N-terminal glycine or by incubation of the cells with the specific myristoyl transferase inhibitor 2-hydroxymyristic acid (HMA, ref. 33) led to the nuclear localization of the fusion protein.

The fact that in these experiments overexpressed LANCL2 was fused to GFP suggested to verify whether similar results could be observed with the untagged protein. In addition, the role of the N-terminal 18 aminoacids in the subcellular localization of untagged LANCL2 was investigated by confocal fluorescence microscopy, as this technique allows to study protein localization in live cells, in real time and with a high sensitivity.

Therefore, HeLa and HEK cells were transiently transfected with various forms of LANCL2 protein: LANCL2 fused to EGFP (LANCL2-GFP), untagged LANCL2, G2A mutagenized LANCL2 (LANCL2-G2A), G2A mutagenized LANCL2 fused to EGFP (LANCL2-G2A-GFP), and short LANCL2 fused to EGFP (LANCL2sh-GFP) (Supplementary Table 1).

**Figure 1:**
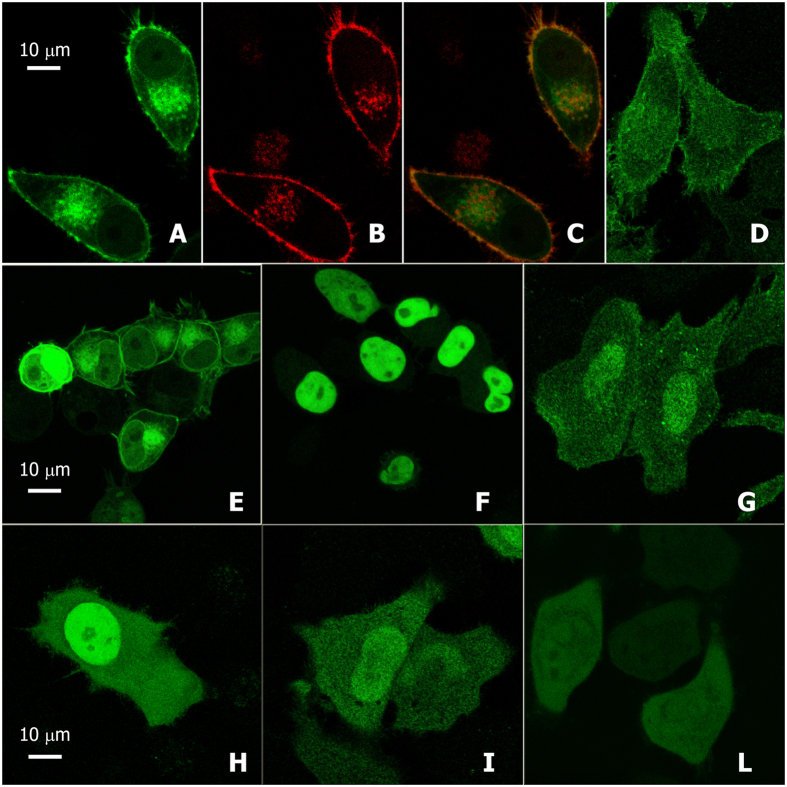
Subcellular localization of overexpressed LANCL2 proteins. HeLa (**A–D,G–L**) or HEK (**E,F**) cells 48 h after transient tranfection with differently tagged LANCL2 forms (Supplementary Table 1). HeLa cells co-transfected with (**A**) LANCL2-GFP and with (**B**) CellLight™ plasmamembrane-RFP, which showed co-localization at the plasmamembrane and juxta-nuclear membrane vesicles in merge panel (**C**). (**D**) The plasmamembrane localization of overexpressed untagged LANCL2 was revealed with monoclonal anti-human LANCL2 mouse antibody (mAb). (**E**) HEK cells overexpressing LANCL2-GFP. Unlike the membrane protein localization of panel (**E**), both the LANCL2-GFP (**F**) and the untagged LANCL2 (panel **G**, LANCL2 revealed with the mAb) lost their membrane localization after treatment with 1 mM of the myristoyl tranferase inhibitor HMA, resulting in an extensive nuclear localization. The non-myristoylatable G2A mutant LANCL2 form showed a similar localization pattern, not only when fused to EGFP (**H**), but also when stained with mAb (untagged mutagenized LANCL2, **I**). Conversely, if the first 18 amino acids were removed, the protein still lost its membrane localization, but shifted to a completely soluble, freely diffusible pattern (**L**).

As shown by the confocal microscope experiments, illustrated in Fig. 1, in HeLa cells, LANCL2-GFP co-localized with a myristoylated plasmamembrane marker (Fig. 1A–C), obtained by expressing the red fluorescent protein fused to the myristoylation/palmitoylation sequence from Lck tyrosine kinase (Molecular Probes, USA). In Fig. 1D, untagged wild-type LANCL2 was revealed with a specific mAb^30^, showing the plasmamembrane localization of LANCL2 also in the absence of tags.

To investigate the effect of de-myristoylation on LANCL2 localization, HEK cells transiently overexpressing LANCL2-GFP were incubated without (control, Fig. 1E) or with 1 mM HMA (Fig. 1F)^33^,^34^. After 24 hours, most of the protein in the HMA-treated cells localized to the nucleus (Fig. 1F) in agreement with previous results^28^, but not in the nucleoli, in contrast with what previously observed.

The nuclear localization of non-myristoylated LANCL2 was confirmed in HeLa expressing untagged LANCL2 treated with HMA and stained with the anti-LANCL2 mAb (Fig. 1G), as well as in cells expressing either mutagenized non-myristoylatable LANCL2-G2A-GFP (Fig. 1H) or untagged LANCL2-G2A (Fig. 1I).

Therefore, no substantial localization differences were observed between various GFP-tagged and untagged LANLC2 recombinant forms (Supplementary Table 1), both in HeLa and in HEK cells. Specifically, while full length myristoylated LANCL2 localized to the plasmamembrane and to cytoplasmic sites (Fig. 1A–E), loss of the myristoyl anchor due to chemical (HMA) or genetic (G2A) inhibition of myristoylation led to the nuclear localization of both the GFP-tagged (Fig. 1F,H, respectively) and the untagged proteins (Fig. 1G,I, respectively).

Accordingly, we explored the presence of nuclear localization signals (NLS) in LANCL2. The cNLS Mapper, a software predictor of NLS^35^, identified two potential NLS sequences in the N-terminal region of LANCL2, with a similar score of 3.6/10: a monopartite NLS (T4-H12) and a bipartite NLS (K7-G37). Indeed, LANCL2-G2A-GFP localized predominantly into the nucleus (Fig. 1H), whereas the truncated form LANCL2sh-GFP, which lacks the N-terminal peptide containing the NLS, showed a homogeneous distribution throughout the cell (Fig. 1L).

### Effects of myristoylation on the intracellular mobility of LANCL2

In order to investigate LANCL2 mobility in live cells, we exploited two different techniques that require the use of fluorescently tagged proteins: fluorescence recovery after photobleaching (FRAP) and fluorescence decay after photoactivation (FDAP).

**Figure 2:**
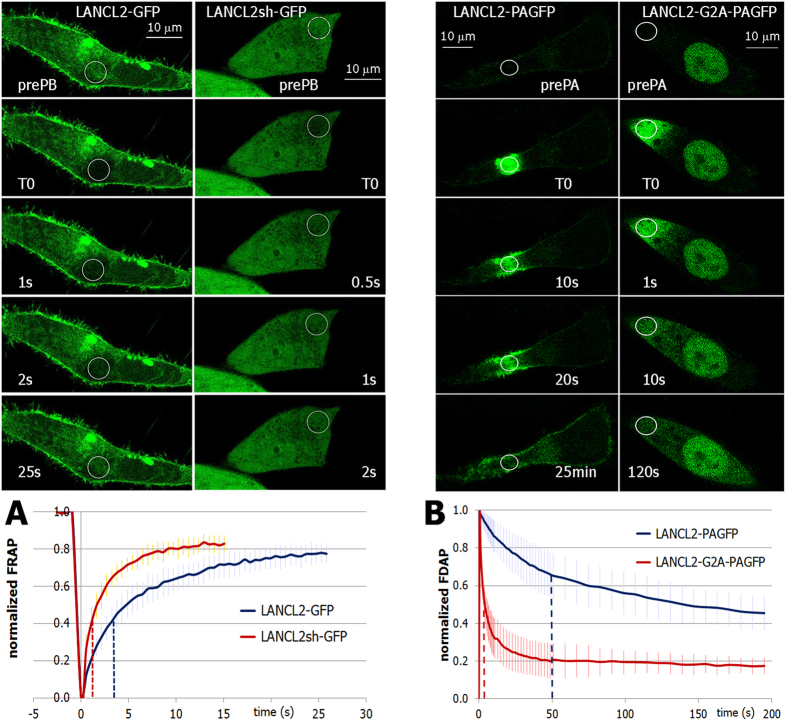
Intracellular mobility of different recombinant forms of LANCL2. (**A**) A representative FRAP experiment. EGFP signal in HeLa cells overexpressing LANCL2-GFP (left column) or LANCL2sh-GFP (right), before (prePB) and at indicated time points after photobleaching (T0). Graph: kinetics of mean FRAP, with s.d., of LANCL2-GFP (blue) and of LANCL2sh-GFP (red trace); the mean half-life constants are indicated in the abscissa as dotted lines. (**B**) A representative FDAP experiment. PAGFP signal in HeLa cells overexpressing LANCL2-PAGFP (left column) or LANCL2-G2A-PAGFP (right), before (prePA) and at different time points after photoactivation (T0). Graph: kinetics of mean FDAP, with s.d., of LANCL2-PAGFP (blue) and of LANCL2-G2A-PAGFP (red trace); the mean half-life constants are indicated in the abscissa as dotted lines.

FRAP explores the mobility of the tagged protein by analyzing the recovery of the fluorescent signal in a specific region of interest (ROI) after permanent photobleaching^36^. To this purpose, HeLa cells were transiently transfected with the full length LANCL2-GFP or with the truncated LANCL2sh-GFP form, and FRAP experiments were performed 48 h after transfection. ROIs were selected in a cytoplasmic area of the cells close to the plasmamembrane avoiding the Golgi and nuclear areas (Fig. 2A).

The average t_½_ calculated for LANCL2-GFP was significantly higher than the t_½_ of LANCL2sh-GFP (3.9 ± 0.9 s and 1.6 ± 0.2 s for LANCL2-GFP and LANCL2sh-GFP, respectively, graph in Fig. 2A; n = 20 cells from three different transfection experiments, p < 0.001).

Then, we also performed photoactivation experiments on LANCL2 fused to photoactivatable GFP (PAGFP). To compare the mobility of wild-type LANCL2 with the non-myristoylatable G2A-mutagenized LANCL2, we transiently expressed LANCL2-PAGFP or LANCL2-G2A-PAGFP in HeLa cells, and photoactivated a circular cytosolic ROI chosen next to the plasmamembrane, in cells of similar shape.

Images acquired at the same time points after photoactivation clearly indicated that the fluorescence decay of LANCL2-PAGFP was much slower than that of LANCL2-G2A-PAGFP (Fig. 2B), as confirmed by calculation of the half time of recovery (t_½_), after fitting values to one phase decay curves^37^: t_½_ values of LANCL2-PAGFP and of LANCL2-G2A-PAGFP were 50 ± 30 s and 3.4 ± 0.75s, respectively (graph in Fig. 2B; =34 cells from four different transfection experiments, p < 0.001).

Moreover, as shown in Supplementary Fig. 2, LANCL2 is capable of rapid lateral diffusion within the plasmamembrane.

Together, these observations indicate that the full length LANCL2 is membrane-associated also in the cytoplasm. When protein myristoylation was prevented by single point mutation, association with the cell membranes was lost, as indicated by its faster free diffusion inside the cell.

### LANCL2 dynamics after ABA treatment

The fact that untagged LANCL2 localized to the cell nucleus when not myristoylated (Fig. 1G–I) suggested to explore whether nuclear translocation of the protein might occur as part of its signaling pathway, following ABA binding. To investigate whether LANCL2 migrated into the nucleus following addition of ABA, we used the full-length form of LANCL2 fused to PAGFP; the finding that LANCL2-GFP and untagged LANCL2 behaved similarly in their nuclear localization upon G2A mutation (Fig. 1H,I, respectively), was taken as a demonstration that the GFP tag did not significantly modify the intrinsic ability of LANCL2 to migrate into the nucleus.

**Figure 3:**
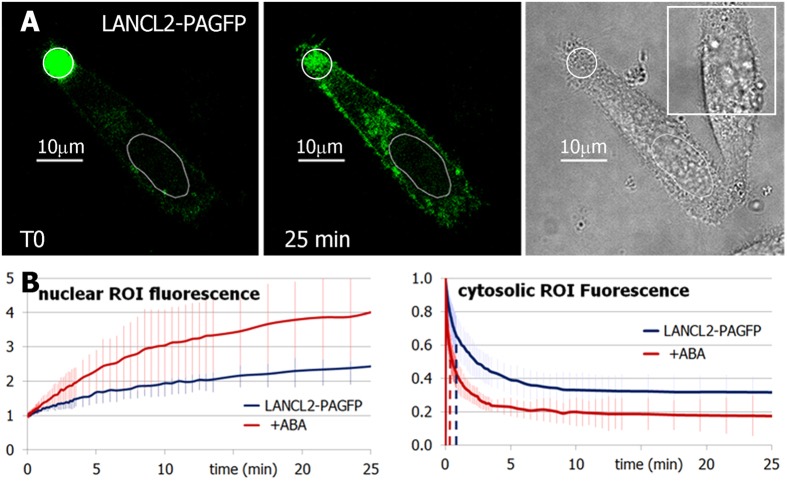
ABA-stimulated LANCL2 nuclear import. (**A**) Representative images of unstimulated HeLa cells transfected with LANCL2-PAGFP, immediately following photoactivation (T0), 25 minutes later and in bright field. ROIs of cytosolic photoactivation (round) and of nuclear analysis (oval) are drawn; a squared ROI, for background fluorescence correction, is indicated on bright field panel (right). (**B**) Graphs: analysis of mean fluorescence increase over time in nuclear ROIs (left), and of fluorescence decay in photoactivated cytoplasmic ROIs (right). HeLa cells were treated (+ABA, red trace) or not (LANCL2-PAGFP, blue trace) with 5 μM ABA, immediately before photoactivation.

At 48 h after transfection with LANCL2-PAGFP, HeLa cells of similar shape and size were subjected to photoactivation in a 8 μm-diameter cytoplasmic ROI, with or without (control) the simultaneous addition of 5 μM ABA; the photoactivation ROI was chosen close to the plasmamembrane, 10–20 μm far from the nearest nuclear membrane, whereas the nuclear ROI covered most of the nuclear area (Fig. 3A). The fluorescent signal from LANCL2-PAGFP rapidly distributed away from the photoactivated region, slowly increasing also inside the nucleus; the dynamics were recorded for 25 min, when a steady-state level of nuclear fluorescence was reached (Fig. 3B, left panel).

At 25 min after photoactivation, the mean nuclear fluorescence, relative to the pre-photoactivation frames, was significantly higher in the ABA-treated cells compared with untreated controls (4.18 ± 1.4 vs. 2.51 ± 0.5, respectively, n = 22 cells from three different transfection experiments, p = 0.005).

Compared with the nuclear enrichment of LANCL2-PAGFP in the absence of ABA, that of PAGFP alone, used as a control, showed a quantitatively similar increase (the plateau of nuclear fluorescence relative to pre-stimulation values was 2.2 ± 0.3), but significantly different kinetics (t½ of 2.2 ± 0.4 and 6.6 ± 1.3 min for PAGFP and LANCL2-PAGFP, respectively, p < 0.001; Supplementary Fig. 3). Thus, the slight increase of nuclear fluorescence of LANCL2-PAGFP in the unstimulated cells could be due to a low extent of ABA-independent, spontaneous, LANCL2 nuclear migration, rather than to non-specific nuclear import of a PAGFP-tagged protein^38^.

Analysis of the decaying fluorescence in the cytoplasmic photoactivated ROI revealed a greater LANCL2 decrease in the ABA-treated than in the untreated cells: the plateau of the fluorescence values relative to the first photoactivated frame and fitted to a single exponential curve, was 0.32 ± 0.09 for control, and 0.22 ± 0.076 for ABA-treated cells (n = 17 cells, p < 0.001; Fig. 3B, right panel). Moreover, the fluorescence decay in the photoactivated cytoplasmic ROI was faster in ABA-treated compared with control cells (the half-life was 50 ± 30 s for control, and 23 ± 13 s for ABA-treated cells; n = 17 cells, p = 0.02).

The higher nuclear content of LANCL2 in the ABA-treated cells, in agreement with the greater and faster LANCL2 decrease in the cytoplasmic photoactivation region, indicates that ABA stimulates LANCL2 migration into the nucleus.

### Role of localization on LANCL2 function

Next, we investigated the role of the myristoylation-dependent membrane localization of LANCL2 in ABA perception/signaling.

ABA binding to LANCL2 eventually results in the stimulation of AC mediated by the βγ subunit complex released from an activated G_i_-protein^5^.

To determine whether LANCL2 directly interacted with the α subunit of a G_i_-protein, we performed FRET experiments, by labelling LANCL2 with a donor fluorophore (EGFP) at the C-terminus (LANCL2-GFP), and the α subunit of a chimeric G_i_ protein, known to be activated by LANCL2^5^, with an acceptor fluorophore (TagRFP, G_i_-RFP). As a negative, non membrane-associated control, we used the EGFP-tagged soluble short form of LANCL2, LANCL2sh-GFP (Supplementary Table 1).

**Figure 4:**
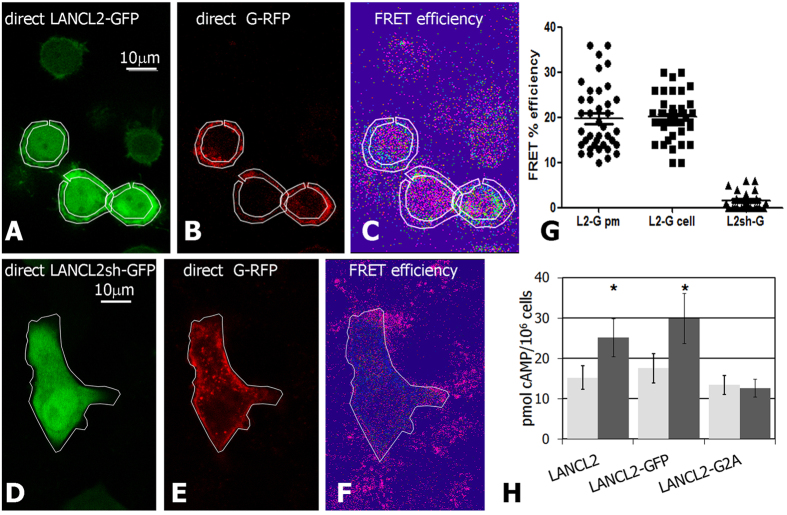
Physical and functional interaction of myristoylated LANCL2 with Gi-protein. FRET analysis between LANCL2-GFP (**A–C**) or LANCL2sh-GFP (**D–F**), and Gi-RFP, in HeLa cells. Representative images of emission of excited donor (GFP-fused proteins: **A,D**), of excited acceptor (G-RFP: **B,E**), and of measured FRET efficiency (**C,F**) are shown in Leica software pseudo colours. Plasmamembrane ROIs are drawn in upper panel, and whole-cell ROIs are shown in lower panel, respectively. (**G**) Graph: the average FRET efficiency between LANCL2 and Gi-protein was not significantly different when calculated in plasmamembranes (LANCL2-G pm) or in intracellular compartments (LANCL2-G cell). Negligible FRET values were measured between soluble LANCL2sh-GFP and G-protein (LANCL2sh-G). (**H**) HeLa cells were transfected with untagged LANCL2 (LANCL2), GFP-tagged LANCL2 (LANCL2-GFP) or untagged mutagenized G2A LANCL2 (LANCL2-G2A). After 48 h from transfection, cells were challenged with 5 μM ABA, and processed for measurement of [cAMP]_i_ levels after 30 s, as described in^15^. Results are expressed as pmol cAMP/10^6^ cells, in untreated (light gray bars) or ABA-stimulated (dark gray bars). *p < 0.02, relative to control, by t test.

Co-transfection of LANCL2-GFP and G_i_-RFP in HeLa cells generated a measurable FRET signal, indicating that the donor (EGFP) and the acceptor (TagRFP) were sufficiently close to allow resonance energy transfer (Fig. 4A–C). Interestingly, FRET between LANCL2 and the G_i_ α subunit was observed not only on plasmamembranes (identified by their intense signal at the border of the cells, and analyzed by manually drawn ROIs), but also in intracellular regions: the average apparent FRET efficiency calculated for the LANCL2-G_i_ couple in plasmamembrane ROIs (20 ± 7%) was similar to the FRET calculated in whole-cell regions (20 ± 5%; n = 40 cells in five different transfection experiments, Fig. 4G). Conversely, the interaction was lost (FRET efficiency 1 ± 2%) when the G_i_-RFP was co-transfected with soluble LANCL2sh-GFP (Fig. 4G).

According to these results, we can infer that the interaction with the α subunit of the chimeric G_i_-protein requires a membrane-associated, myristoylated, LANCL2, the close proximity between LANCL2 and G_i_ occurring both on the plasmamembrane and on intracellular membranes.

We then compared the ABA-binding ability of purified recombinant LANCL2 (LANCL2-gst) and of its truncated short form (LANCL2sh-gst). Both forms were cleaved from the GST tag (Supplementary Table 1), produced as described in^6^, but cleaved in the absence of DTT, a more physiological condition leading to a higher binding affinity than previously observed. Indeed, specific and saturable [^3^H]ABA binding occurred to both proteins with similar affinities (Kd values were 8.9 ± 1 μM and 10.6 ± 1.4 μM for LANCL2 and LANCL2sh, respectively, from three different experiments).

Thus, the 18 aminoacids N-terminal stretch of LANCL2, which includes the myristoylation site, is not necessary for ABA binding.

Finally, we explored the ability of different LANCL2 forms to trigger the ABA-signaling pathway, by measuring the increase of the intracellular concentration of cAMP ([cAMP]_i_) in transfected HeLa cells following addition of ABA (as described in^15^).

HeLa cells transfected either with untagged LANCL2, or with LANCL2-GFP, or with the untagged mutagenized LANCL2 (LANCL2-G2A) were incubated with 5 μM ABA and the [cAMP]_i_ was measured after 30 s. The [cAMP]_i_ increased in HeLa cells overexpressing untagged LANCL2 (165 ± 19% over basal, unstimulated values, p < 0.02) or LANCL2-GFP (170 ± 21%, p < 0.02). Conversely, the [cAMP]_i_ was not significantly modified compared to basal values in cells transfected with the non-myristoylatable LANCL2-G2A (94 ± 17%, p = 0.6), Fig. 4H.

Therefore, LANCL2 myristoylation and a functional interaction between LANCL2 and the chimeric G_i_ α subunit (Fig. 4H) are essential for the ABA-triggered activation of adenylate cyclase, both requirements being met at the plasmamembrane and at internal membranes in the cytoplasm.

Conversely, binding of ABA does not require association of LANCL2 with the plasmamembrane, as it is not significantly affected by the truncation of 18 N–terminal aminoacids. This observation suggests that an unmyristoylated, and thus freely-diffusible, LANCL2 could be able to bind ABA and activate other, cAMP-independent, signaling pathways.

## Discussion

Here, we demonstrated that N-terminal glycine myristoylation causes LANCL2 localization to the plasmamembrane and to cytoplasmic membrane vesicles as well. Interestingly, evidence has recently been provided of non-canonical subcellular sites for G protein activation: these include endosomal localization of functionally active GPCR^39^,^40^,^41^, and the interaction of the GIV/Girdin protein, a non-receptor guanine nucleotide exchange factor, with heterotrimeric G proteins at the Golgi^42^,^43^ and at still undefined intracellular sites^44^. The effectiveness of N-myristoylated LANCL2 in coupling to G_i_ physically and functionally (FRET efficiency and [cAMP]_i_ increase, Fig. 4) also when associated to internal membrane vesicles represents a further example of G-protein mediated signal initiation at non-canonical intracellular sites.

The finding of nuclear localization of LANCL2 after exposure of the cells to ABA (Fig. 3) suggests a possible role for LANCL2 in ABA-triggered transcriptional events. Indeed, stimulation by ABA of the transcription of several cytokines regulating the proliferation of human mesenchymal and hemopoietic stem cells has been previously observed^18^,^19^, and ABA-induced transcriptome variations are well established in plants^45^.

A still unanswered question is whether demyristoylation of native LANCL2 (e.g., via specific enzymes or limited proteolysis targeting the N-terminus) is required for nuclear import and whether ABA binding itself can boost demyristoylation. So far, we were unable to identify any demyristoylated form of LANCL2, which could however escape detection for several reasons (e.g., transient nature due to rapid nuclear clearance, or low specificity of anti-myristoyl anchor antibody).

The facts that LANCL2 is intracellular and that its hormone ligand ABA is an anion at physiological pH values, and thus unable to diffuse across the membrane lipid bilayer, imply that a transmembrane transport is required to allow extracellular ABA to reach its receptor. Indeed, influx of ABA has been recently demonstrated to occur through the transmembrane bicarbonate/chloride exchanger 1 (AE1, band 3 protein) in erythrocytes^30^. Moreover, the bidirectional nature of ABA transport across the plasmamembrane may also support paracrine mechanisms, already observed with this hormone^9^,^10^,^17^,^18^,^19^,^21^,^22^, based upon release of ABA from one cell and its perception/signaling in a neighboring cell. In any case, the fact that ABA enters the cells through a transmembrane transporter is unique among animal hydrophilic hormones, which bind to the extracellular domain of integral membrane receptors.

In conclusion, this study highlights some non-canonical features of the mammalian ABA receptor: i) LANCL2 is a membrane-bound, but not an integral membrane protein, whose myristoylation allows coupling to a G_i_ protein and activation of adenylate cyclase; ii) upon ABA-binding LANCL2 can translocate into the nucleus, to our knowledge an unprecedented feature for a membrane-associated, G-protein coupled, receptor.

The diversified LANCL2 intracellular behavior might account for the emerging multifunctionality of LANCL2^46^, for the heterogeneity of cell targets and for the pleiotropy of ABA-stimulated regulatory effects in these cells, impacting on fundamental systemic functions^7^,^8^,^9^,^10^,^15^,^16^,^17^,^18^,^19^,^47^.

These results broaden our knowledge on the mechanisms underlying hormonal receptor-G protein interaction in animals, by providing the first observation of a non-transmembrane G protein-coupled receptor capable of hormone-induced nuclear translocation. Thus, LANCL2 appears to be uniquely endowed with two key features (G protein-coupling and nuclear translocation), each one typical of a different family of animal hormone receptors, i.e. peptide and steroid hormone receptors, respectively.

Conservation of ABA as a stress hormone regulating cell responses to environmental stimuli in plants, lower Metazoa (sponges and hydroids)^48^,^49^ and mammals, suggests its origin in a common precursor to animals and plants. This ancient evolutionary root may indeed place this hormone in a category of its own compared to the other animal hormones.

## Materials and Methods

### Reagents and antibodies

The mouse monoclonal antibody against human LANCL2 (LANCL2 mAb) was produced by Dr. C. Fresia at Molecular Biotechnology Center (MBC), Turin (Italy), as previously described^30^. Primary antibody anti-human vinculin was a kind gift of E. Turco (MBC, Turin). HRP-conjugated secondary antibodies were from Santa Cruz Biotechnology (Santa Cruz, CA); Alexa-488-conjugated ones were from Thermo Fisher Scientific (NY, USA). 2-hydroxymyristic acid (HMA), tissue culture media and supplements were purchased from Sigma-Aldrich (Milan, Italy). HMA, dissolved in chloroform and dryed out with N_2_, was resuspended at a 10 mM concentration in 1% fatty acid free BSA in HBSS, through overnight shaking^33^,^34^.

### Cell cultures and transfection

HeLa, HEK-293, MDA-MB-468, MDA-MB-239 cells were cultured in Dulbecco’s modified Eagle’s complete cell culture medium (DMEM with 10% fetal bovine serum, 50 U/ml penicillin, 50 μg/ml streptomycin). Cells were seeded at 15.000/cm^2^, on 25 mm glass coverslip Menzel-Glaser thickness N1.5 (Tecnovetro SRL, Monza, Italy), on 8-wells chambered coverglasses (Lab-Tek, Nunc, Thermo Fisher Scientific, NY, USA), or on 35 mm Petri dishes 24 hours before transfection, and maintained in a humidified 5% CO_2_ atmosphere at 37 °C.

Cells were transfected with the calcium-phosphate method^50^, adding to the fresh complete medium a 10% volume of transfection reagent composed of Hepes Buffered Saline, 100 mM CaCl_2_ and 30 μg/ml endotoxin-free DNA (prepared with EndoFree Plasmid Midi Kit, QIAGEN, following manufacturer instructions). Medium was replaced after 12–16 h.

The ready-to-use construct CellLight^®^ Plasma Membrane-RFP (Molecular Probes, Thermo Fisher Scientific, Waltham, MA USA) was transfected into cells 24 h after LANCL2-GFP transfection, using BacMam 2.0 technology; it was obtained by expressing the red fluorescent protein (RFP) fused to the myristoylation/palmitoylation sequence from Lck tyrosine kinase, providing accurate and specific targeting to cellular plasmamembrane.

### Western Blot

Western blot analyses were performed following standard methods, as previously described^28^: 5–50 μg of different cell lysates (1% SDS, 5 mM EDTA, 10 mM DTT, 1:200 of protease inhibitors cocktail, Sigma-Aldrich) were loaded on a 10% polyacrylamide gel.

### Recombinant proteins and [^3^H]ABA binding

Expression and purification of the recombinant proteins were performed as previously described^5^. BL21 (DE) *E. coli* cells utilized were grown in Luria–Bertani medium (Difco, BD Italia, Roma, Italy).

The release of LANCL2-gst or of LANCL2sh-gst (Supplementary Table 1) was achieved with the PreScission Protease (GE Healthcare), by incubating the GSH-Sepharose-bound fusion protein for 16 h at 4 °C following manufacturer instruction, but without DTT: the protease cleavage left 9 amino acids at the N-terminus of the protein, considered in the Mw calculations.

To compare specific [^3^H]ABA binding of recombinant LANCL2-gst and LANCL2sh-gst produced in *E. coli*, we proceeded as previously described in^6^.

[^3^H]ABA binding experiments to intact MDA-MB-468 and MDA-MB-231 cells, were performed as previously described in^15^.

### Immunofluorescence and fluorophores

HeLa or HEK-293 cells were grown on 18- or 25-mm diameter coverslips, previously coated with poly-L-lysine (Sigma-Aldrich), for 24 hours before transfection, 2-HMA treatment or fixation. Specimens were treated for fixation at RT for 10 min with para-formaldehyde (4% in PBS), then permeabilized for 8 min with 0.1% Triton X100, blocked for 10 min with 3% BSA, incubated for 1 hour with anti-LANCL2 mAb 5 μg/ml in BSA 3%, and finally stained for 30 min with 10 μg/ml Alexa Fluor 488 secondary antibody (Thermo Scientific). Mounting medium was Mowiol +2.5% DABCO (Sigma-Aldrich).

For live analysis cells were maintained in complete DMEM without phenol red, supplemented with 20 mM Hepes.

Excitation/emission maxima of fluorophores utilized were: Alexa 488 495/519, EGFP 488/507, PAGFP 504/517, TagRFP and CellLight^®^ -RFP 555/584.

Localization images were obtained using a Leica TCS SP2 confocal microscope (Leica Microsystem, Heidelberg, Germany) equipped with argon/He-Ne laser sources and Leica HCX PL APO CS 63.0 X N.A1.40 oil objective.

To exclude that the EGFP-fusion could affect subcellular distribution, we treated HeLa cells overexpressing untagged LANCL2 with the N-myristoyl transferase inhibitor 2-hydroxymyristic acid (HMA), then stained the protein with a specific anti-LANCL2 mAb; as a further control to ensure that no intracellular morphological changes affecting LANCL2 localization could have been induced by the HMA treatment^51^,^52^, we used the mutagenized form of LANCL2 bearing the G2A mutation (therefore not susceptible to undergo myristoylation), fused to EGFP.

### cDNA constructs

cDNA encoding human LANCL2 was subcloned from previously available constructs^6^ into the following expression vector: pEGFP-N, pcDNA3.1^+^, pPAGFP-N1, pGEX-6P-1. When compatible, restriction enzyme sites were used for subcloning; otherwise, and for truncated LANCL2 forms (LANCL2sh-gst, LANCL2sh-GFP), specific primers containing the desired restriction enzyme sites were designed for PCR amplification from plasmid (TibMolBiol, Genova, Italy). The G2A site-specific mutagenesis was obtained with the QuickChange Lighting Site-Directed Mutagenesis kit (Agilent Technologies; Santa Clara, CA), following manufacturer’s instructions, applied on the corresponding/analogous/appropriate vector.

EGFP and PAGFP tags were fused at the C-terminus of LANCL2 protein forms.

The EGFP coding sequence contained an A207K mutation to eliminate potential dimerization of the EGFP moiety^53^.

The α subunit of the chimeric G_i_ protein^5^ was subcloned into pTagRFP-C (Evrogen, Moscow, Russia), thus resulting N-terminally fused to TagRFP monomeric protein.

All cDNA constructs (Supplementary Table 1) were confirmed by sequencing (TibMolBiol).

### FRAP and photoactivation experiments

HeLa cells were transiently transfected with LANCL2-GFP or LANCL2sh-GFP for FRAP experiments, or with LANCL2-PAGFP or LANCL2-G2A-PAGFP for FDAP experiments, and processed 48 h after transfection. Images were acquired by a Nikon A1 confocal microscope (Nikon Corporation, Tokio, Japan), equipped with a 60 X PlanApo oil immersion objective (NA 1.40). All images, corrected for fluorescence background, were analysed with ImageJ 1.48v (Wayne Rasband, Nat. Inst. of Health, USA), and the obtained data with the Excel software (Microsoft).

For FRAP experiments, the EGFP fluorescence emission was bleached in a 6 μm-diameter ROI for 0.12 s, with the 405 nm laser line, zooming to the ROI to minimize the photobleaching duration^54^. Fluorescence recovery was followed for 120 s, exciting the sample with the 488nm Argon Laser-line.

At each time point the mean fluorescence value (F_t_) in the bleached ROI was analysed as described^37^,^55^: each ROI F_t_ was normalized for the F_t_ of a ROI not subjected to photobleaching, to correct for photobleaching of EGFP due to the imaging procedure. Photobleaching-corrected F_t_ values were subtracted of first post-photobleaching values and normalized for the mean fluorescence value measured before photobleaching.

Fluorescence recovery traces were fitted with a single exponential equation (GraphPad Prism, version 5.01 for Windows, California, USA), as follows:


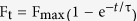


which allowed to calculate the half-time constant (t_½_), the time it takes for the fluorescence to recover to 50% of the asymptote (plateau) intensity (F_max_), as t_½_  = τ*ln2.

For each experimental condition, all curves were averaged and t_½_ values of different populations were compared using Student’s t-test.

ROIs of different size were analyzed, yielding similar results.

Photoactivation was performed by exciting the PAGFP with the 405 nm laser line inside a 8 μm-diameter ROI for 0.063 s. The fluorescence decay in the photoactivated region was imaged for 3 min for fast-diffusing LANCL2-G2A-PAGFP, and for 25 min for LANCL2-PAGFP, with 488nm laser line.

As control, we used PFA-fixed HeLa cells expressing LANCL2-PAGFP to monitor the potential photobleaching of PAGFP during the observation time.

No bleaching was observed in fixed cells in the time interval of our experiments, nor in ROIs chosen in living transfected HeLa cells after whole-cell photoactivation (data not shown).

After photoactivation, the mean fluorescence value (F_t_) of the ROI at each time point was corrected for the fluorescence measured before the photoactivation; then it was normalized to F_t0_, where t0 is the first frame after the photoactivation.

Data were fitted with a single exponential decay equation, according to


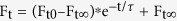


where F_t∞_ is the normalized fluorescence value at infinite time. The half-life (t_½_) for each experimental condition was averaged, and the different populations were compared using Student’s t-test.

### FRET imaging and analysis

Fluorescence (or Förster) Resonance Energy Transfer (FRET) allows the study of protein interactions, measuring a non-radiative transfer of energy from an excited state or a fluorophore (donor) to a different fluorophore (acceptor), over distances comparable to the size of proteins^56^,^57^. As stated by the non-invasive method of sensitized emission (SE) applied to live specimens, following donor excitation, FRET results in a decrease in donor emission with a simultaneous increase in acceptor emission, due to a dipolar interaction between the molecules^58^.

The expression of EGFP and TagRFP tagged proteins in HeLa cells grown in 8-wells chambered coverglasses (Lab-Tek, Nunc, Thermo Scientific) was visualized 2 days after transfection by confocal microscopy using a Leica TCS SP5 AOBS (Leica Microsystem, Heidelberg, Germany) equipped with an oil immersion objective 63x/1.4 NA. Light collection configuration was optimized according to the combination of chosen fluorochromes, selecting the spectral windows by the acousto-optic beam splitter of the Leica SP5 scanning head.

The Leica “LAS AF” software package was used for image acquisition. FRET efficiency (FE) was measured using the Sensitized Emission method^57^. Briefly, measurements were performed by detection of the fluorescent signals of the donor, FRET and acceptor in a line by line sequential scan acquisition, obtained with the Leica Microsystem “FRET SE Wizard” software. The same process was applied to donor-only and acceptor-only reference specimens, on cells chosen to visually display mean fluorescence intensity. From the images obtained, we calculated the calibration coefficients required to correct for excitation and emission crosstalk.

FE was calculated in plasmamembrane-representing or in whole-cell-including ROIs, for 8–15 cells in each experiment, according to FE = (B − βA − γC)/C, where A, B, C are the intensities of the donor, FRET and acceptor emissions and β and γ are the calibration factors correcting for donor cross-talk and acceptor cross-excitation, respectively.

### Statistical Analysis

Data were compared using an unpaired Student’s t test. Statistical significance was set at P-value < 0.05. Statistical analysis was performed using the GraphPad Prism Software, or Microsoft Excel software.

## Additional Information

**How to cite this article**: Fresia, C. *et al*. G-protein coupling and nuclear translocation of the human abscisic acid receptor LANCL2. *Sci. Rep.* **6**, 26658; doi: 10.1038/srep26658 (2016).

## Supplementary Material

Supplementary Information
