# Supplementary material for: G-protein coupling and nuclear translocation of the human abscisic acid receptor LANCL2
Source: Sci Rep. 2016 May 25;6:26658. doi: 10.1038/srep26658 (PMC4879523; doi:10.1038/srep26658)
Supplement: Supplementary Information [file srep26658-s1.docx]

**SUPPLEMENTARY MATERIALS FOR**

**G-protein coupling and nuclear translocation of the human abscisic acid receptor LANCL2**

**Authors**: Chiara Fresia^1^*, Tiziana Vigliarolo^1^, Lucrezia Guida^1^, Valeria Booz^1^, Santina Bruzzone^1^, Laura Sturla^1^, Melody Di Bona^2^, Mattia Pesce^2^, Cesare Usai^3^, Antonio De Flora^1^, Elena Zocchi^1^

**SUPPLEMENTARY TABLE**

| **Protein form** | **N-terminus** | | **C-terminus** | **aa** |
| --- | --- | --- | --- | --- |
| **LANCL2** (L2) |  | **M**GET**M**SKRLKLHLGGEAE**M**EE~ 1 5 19 | - | 450 |
| LANCL1 |  | **M**AQ~ | - | 400 |
| LANCL3 |  | **M**DTK~ | - | 388 |
| LANCL2-G2A |  | **M**AET**M**SKRLKLHLGGEAE**M**EE~ | - | 450 |
| LANCL2-gst | GST (cleaved) | **M**GET**M**SKRLKLHLGGEAE**M**EE~ | - | 459 |
| LANCL2sh-gst |  | **M**EE~ | - | 441 |
| LANCL2sh-GFP |  | **M**EE~ | EGFP | 676 |
| LANCL2-GFP |  | **M**GET**M**SKRLKLHLGGEAE**M**EE~ | EGFP | 694 |
| LANCL2-G2A-GFP |  | **M**AET**M**SKRLKLHLGGEAE**M**EE~ | EGFP | 694 |
| G_q/i_ chimera | TagRFP |  | - | 597 |

**Supplementary Table 1**

List of the various recombinant proteins investigated in this study, their N-terminal/C-terminal tags, and total aminoacids number

**SUPPLEMENTARY FIGURES**

**
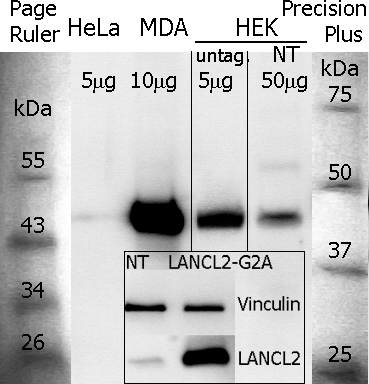
**

**Supplementary Figure 1a**

**SDS-PAGE electrophoresis of LANCL2 proteins.**

Western blot analysis of various human cell types, transfected or not with full length recombinant LANCL2, revealed with the primary mAb anti-human LANCL2 (30).

Different protein standards were used (Fermentas Page Ruler™ Plus on the left and BioRad Precision Plus Protein™ on the right). LANCL2 proteins migrate at a calculated Mw of 49.7 kDa. Cell extracts: HeLa (5µg), MDA-MB-468 (MDA, 10µg), HEK-293, transfected (5µg, untag.) or not (50µg, NT) with untagged form of LANCL2.

Inset: Western blot analysis of HEK-293 lysates immunostained with anti-LANCL2 mAb, and anti-Vinculin mAb for normalization. The mobility of endogenous LANCL2 (in HEK transfected with the empty pcDNA3.1 vector: NT) is the same as that of the overexpressed, untagged, G2A mutagenized form (LANCL2-G2A).

**Supplementary Figure 1b**

**Unprocessed original scans of the blots in supplementary Figure 1a**


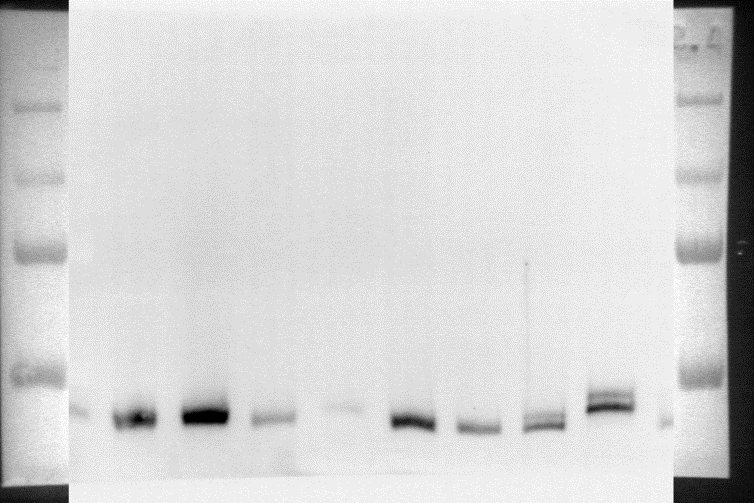

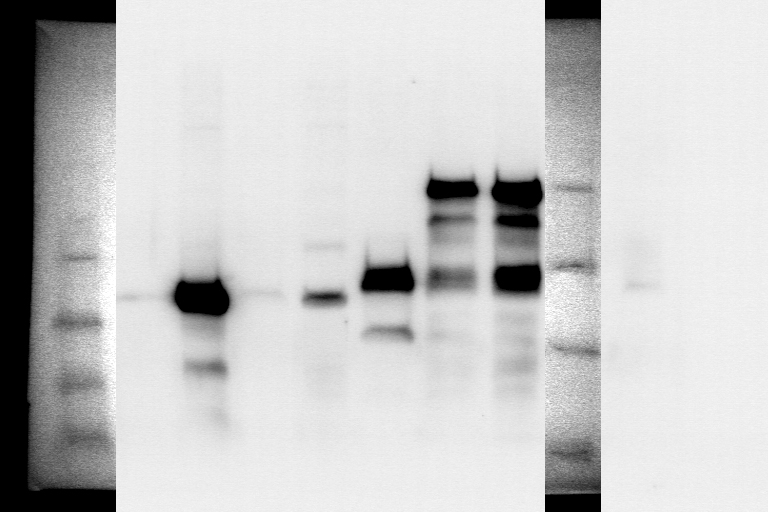


**Left panel**: main parts of supplementary fig. 1a, plus MW markers;

**Right panel**: HEK-untagged, lane


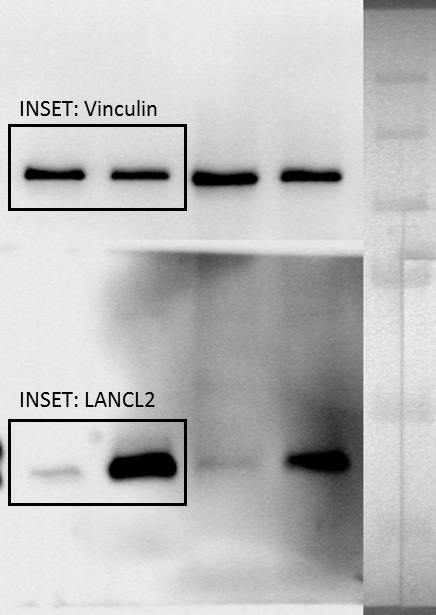


Original scan of supplementary Figure 1a, **inset**


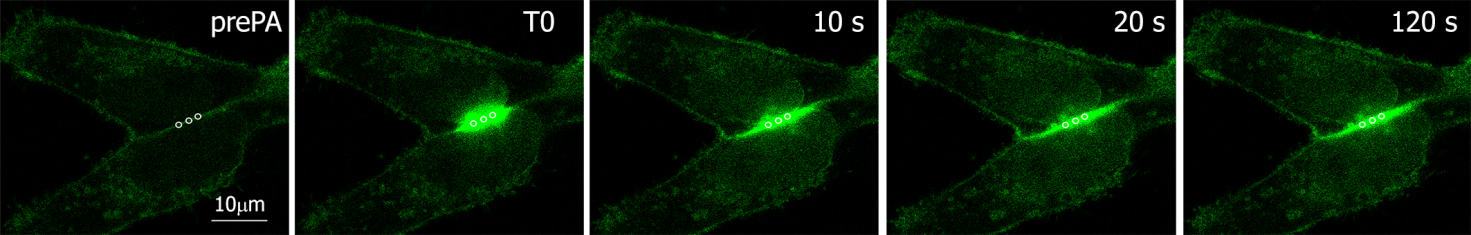


**Supplementary Figure 2**

**Mobility of photoactivated LANCL2-PAGFP along the plasmamembrane of HeLa cells**

Representative cell images from a plasmamembrane “single-point” photoactivation experiment of HeLa cells overexpressing LANCL2-PAGFP.

PAGFP signal before (prePA) and at different time points after photoactivation (indicated as T0).


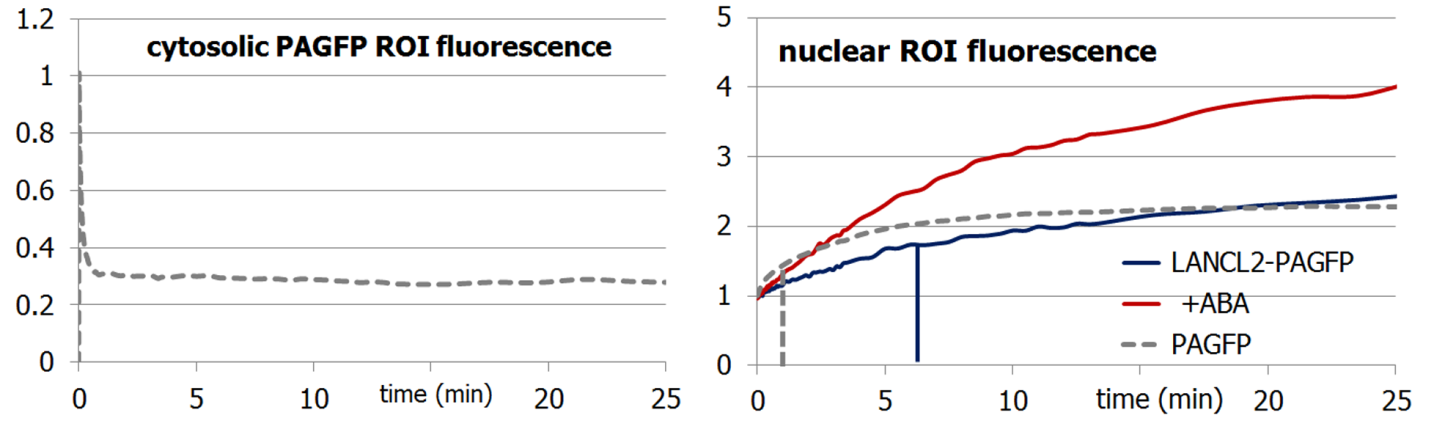


**Supplementary Figure 3**

**Spontaneous PAGFP nuclear diffusion**

25 min analysis of mean fluorescence decay of photoactivated cytoplasmic ROIs (left), and of mean fluorescence increase over time in nuclear ROIs (right). In the nuclear ROI fluorescence graph, traces of fluorescence of LANCL2-PAGFP in HeLa cells treated or not with ABA (as in Fig 2) are reported to facilitate comparison; the mean half-life constants of LANCL2-PAGFP and PAGFP are indicated in the abscissa as vertical lines.
